# Evaluation the Effectiveness of Abridged IMNCI (7-Day) Course v Standard (11-Day) Course in Pakistan

**DOI:** 10.1007/s10995-021-03276-3

**Published:** 2021-10-20

**Authors:** Shabina Ariff, Kamran Sadiq, Uswa Jiwani, Khalil Ahmed, Khadija Nuzhat, Shakeel Ahmed, Qamruddin Nizami, Iqtidar A. Khan, Nabeela Ali, Sajid Bashir Soofi, Zulfiqar A. Bhutta

**Affiliations:** 1 Department of Pediatrics and Child Health, Aga Khan University, Karachi, Pakistan; 2 Center of Excellence in Women and Child Health, Aga Khan University, Stadium Road, Karachi, 74800 Pakistan; 3 John Snow Inc (JSI), Arlington, USA

**Keywords:** IMNCI, Neonatal health, Child health, Training

## Abstract

**Background:**

The conventional IMCI training for healthcare providers is delivered in 11 days, which can be expensive and disruptive to the normal clinical routines of the providers. An equally effective, shorter training course may address these challenges.

**Methods:**

We conducted a quasi-experimental study in two provinces (Sindh and Punjab) of Pakistan. 104 healthcare providers were conveniently selected to receive either the abridged (7-day) or the standard (11-day) training. Knowledge and clinical skills of the participants were assessed before, immediately on conclusion of, and six months after the training.

**Results:**

The improvement in mean knowledge scores of the 7-day and 11-day training groups was 31.6 (95% CI 24.3, 38.8) and 29.4 (95% CI 23.9, 34.9) respectively, *p* = 0.630 while the improvement in mean clinical skills scores of the 7-day and 11-day training groups was 23.8 (95% CI: 19.3, 28.2) and 23.0 (95% CI 18.9, 27.0) respectively, *p* = 0.784. The decline in mean knowledge scores six months after the training was − 12.4 (95% CI − 18.5, − 6.4) and − 6.4 (95% CI − 10.5, − 2.3) in the 7-day and 11-day groups respectively, *p* = 0.094. The decline in mean clinical skills scores six months after the training was − 6.3 (95% CI − 11.3, − 1.3) in the 7-day training group and − 9.1 (95% CI − 11.5, − 6.6) in the 11-day group, *p* = 0.308.

**Conclusion:**

An abridged IMNCI training is equally effective as the standard training. However, training for certain illnesses may be better delivered by the standard course.

**Supplementary Information:**

The online version contains supplementary material available at 10.1007/s10995-021-03276-3.

## Significance

We wanted to objectively assess the productivity of a short IMNCI course to improve the coverage of IMNCI in the country and reduce cost. We showed that the seven day training is effective.

## Introduction

Approximately half of the world’s under-five and neonatal deaths occur in only five countries: India, Nigeria, Ethiopia, the Democratic Republic of Congo and Pakistan (UNICEF, 2019). Although the global under-five and neonatal mortality rates have decreased by more than half since 1990, thousands of childhood deaths still occur in southern Asia and sub-Saharan Africa every day (UNICEF, 2019). A substantial proportion of the decline in childhood mortality was achieved by addressing the two major preventable causes of deaths, pneumonia and diarrhea (Jin et al., 2018). Despite these efforts, both pneumonia and diarrhea still contribute significantly to child mortality (UNICEF, 2019).

To address this global burden, the World Health Organization (WHO) and the United Nations Children’s Fund (UNICEF) developed a strategy called the Integrated Management of Childhood Illness (IMCI). IMCI encompassed a consolidated approach for managing the five most common causes of death in under-five children; pneumonia, diarrhea, measles, malaria and malnutrition (World Health Organization & UNICEF, 1999). Care for newborns younger than 7 days was added to the program in 2003 and the strategy came to be known as IMNCI in many countries (Maternal and Child Survival Program, 2019). Pakistan’s Ministry of Health formally endorsed IMCI in 1998 and it was implemented in the country in 2000 to address the high infant and child mortality rates (World Health Organization Regional Office for the Eastern Mediterranean, 1996).

The effectiveness of IMCI in improving health care workers’ ability to assess and manage the sick child has been well documented in literature (Amaral et al., 2004; Chopra et al., 2005). A systematic review reported that IMCI training and implementation may also cause a reduction in child and infant mortality (Gera et al., 2016). Regardless of the demonstrated advantages of the strategy, its implementation has been met with several challenges, including shortage of trained medical staff, essential drugs, and equipment, weak health systems, and budget allocations (Ahmed et al., 2010; Pradhan et al., 2013; Titaley et al., 2014).

In low-middle income countries, the expense associated with the 11-day IMCI case-management training is a significant problem for scaling up the strategy (Mushi et al., 2011). Additionally, the absence of participating healthcare workers from their clinical obligations leads to disruptions, scarcity of medical staff, and compromised care for the patients. An abridged training has been identified as an alternative to overcome the financial and operational difficulties (Goga & Muhe, 2011). However, before implementing an abridged IMNCI training course, its effectiveness must be evaluated. The objective of this study was to compare the effect of a short 7-day IMNCI training with the standard 11-day training on healthcare providers’ ability to assess and classify illness and take appropriate action according to the IMNCI protocol. We hypothesize that there will be no significant differences in the assessment scores of providers who receive the shorter training and providers who receive the standard duration of training.

## Methodology

### Study Design and Setting

A quasi-experimental study was carried out from April 2009 to October 2009 in districts Sukkur and Khairpur from Sindh and districts Rawalpindi and Islamabad from Punjab. Two IMNCI training sessions were conducted in each province; a standard WHO 11-day training and an abridged 7-day training that was adapted from the standard course. Blinding of the participants and trainers was not feasible because the same facilitators conducted the training for both groups.

### Study Population

Healthcare providers from public sector facilities were nominated by the District Health Officers while those from private facilities were recruited through self-selection after invitation. Providers were eligible to participate in the training if they: (i) worked at first-level health facilities such as hospital outpatient services, health centers, dispensaries, and clinics, (ii) 3 to 4 years of experience in providing clinical care to infants and children, and (iii) did not receive IMNCI in-service training previously. The providers were non-randomly divided into two groups to receive either the 7-day or the 11-day training. The two groups were comparable in terms of cadre (physicians or other healthcare providers) and the health sector they worked in (public or private). The other providers included registered nurses, midwives, health technicians, and lady health visitors (LHVs). LHVs are an important cadre of healthcare workers who provide reproductive and child healthcare at the household and facility levels. They undergo one year of training in midwifery and one year in pediatric and tropical diseases (Ariff et al., 2010). They are stationed at the Basic Health Units, Rural Health Centers, and Tehsil Headquarter Hospitals and provide antenatal, postnatal and immediate newborn care, and facilitate referral to higher centers (Ariff et al., 2010).

### IMNCI Case-Management Training

The training was held at two public sector tertiary care hospitals: the Pakistan Institute of Medical Sciences (PIMS) Hospital, Islamabad for the Islamabad and Rawalpindi participants, and the Civil Hospital, Sukkur for the Khairpur and Sukkur participants. Sessions were facilitated by WHO-certified master trainers from the Department of Paediatrics and Child Health at the Aga Khan University. To ensure uniformity and reduce environmental bias, sessions were led by the same team of facilitators for both groups. The training consisted of eight modules that were delivered via classroom activities and hands-on clinical practice sessions as recommended by WHO. The clinical practice sessions were conducted in the outpatient clinics and inpatient wards of the two hospitals. The participants observed and practiced the assessment, classification, and treatment of sick children according to the IMNCI protocol. They were also provided with the IMNCI recording forms to fill their patients’ information.

The same teaching materials, tools, curriculum, and training methodologies were used for both groups. The course was only redesigned and shortened in terms of the time allocated to each classroom activity and clinical practice session. A description of the training and the time spent on each activity in both groups is presented in Supplement 1.

### Pre- and Post-Training Assessment

All assessments were conducted at PIMS Hospital, Islamabad and Civil Hospital, Sukkur. Participants’ knowledge and clinical skills were assessed at the beginning and immediately after training. The level of knowledge was measured by administering a written test comprising 25 MCQs. Each correct answer was given a score of two and incorrect answers were marked zero. The questions tested the providers’ knowledge on seven common childhood ailments: possible bacterial infection (PBI), acute respiratory infection (ARI), diarrhea, malaria, ear infection, malnutrition, and anemia, along with breastfeeding problems and counseling.

The clinical skills were assessed in two ways. First, the providers were directly observed in the hospital outpatient and inpatient departments by the facilitators and evaluated according to the IMNCI standard skills observation checklist. Second, the providers were shown 12 video case studies and asked questions on the clinical scenarios presented in the videos for a maximum score of 33. The scenarios covered the seven clinical conditions mentioned above. Participants were expected to appreciate IMNCI signs (for example, sub-costal recessions, stridor, and nasal flaring in respiratory distress, sunken eyes, lethargy, and a slow return to normal after skin pinch in dehydration, edema in malnutrition, and purulent discharge and mastoid swelling in ear infection), count respiratory rate, classify the level of dehydration, nutritional status, ear problem, and respiratory symptoms, and recognize proper attachment and suckling during breastfeeding.

Six months following the IMNCI training, a re-assessment was carried out to evaluate and compare the retention of knowledge and clinical skills between the two groups. The re-assessment was done with the same MCQs and video-based tools that were used at the beginning and end of the training. The facilitators also directly observed the participants’ case-management abilities and their clinical skills.

### Outcome Measures

The primary outcomes were the improvement and retention of knowledge and clinical skills of the two groups. Improvement was defined as the difference between the means of the pre-training and immediate post-training assessment scores. Retention was defined as the difference between the means of the post-training and re-assessment scores. The secondary outcomes were the differences in mean scores for each of the childhood illnesses assessed between the two groups.

### Sample Size Calculation

Assuming an α error of 5% and power of 80%, a minimum sample size of 40 participants in each group was required to detect a 10% difference in post-training test scores (Kumar et al., 2009).

### Data Analysis

Data were analyzed using SPSS version 15. Only the scores of the participants who were re-assessed at 6 months were used for the analysis. The scores were reported as mean percentages with standard deviations. Unpaired t-tests were used to compare the scores between the two groups. A *p* < 0.05 was considered statistically significant.

### Cost Estimation

The total expenses of the training were calculated by including the travel and accommodation costs of the facilitators and the participants, facilitation fees, cost of refreshments, stationery, and teaching materials. Because all training sessions were conducted in public sector hospitals, there were no additional expenses for logistic arrangements.

### Ethical Considerations

The study was approved by the Ethics Review Committee of the Aga Khan University and was performed according to the ethical standards of the 1964 Declaration of Helsinki and its later amendments. Verbal informed consent for partaking in the study was obtained from all participants before including them in the study.

## Results

A total of 104 healthcare providers participated in the study. 46 (44.2%) providers received the 11-day and 58 (55.8%) received the 7-day training. Their socio-demographic characteristics are shown in Table 1. Of all the participants, 61 were re-assessed 6 months after the initial training, 32 (52.4%) from the 11-day and 29 (47.5%) from the 7-day training groups. Their characteristics are presented in Table 2. The characteristics of the remaining 43 participants who were not re-assessed at 6 months are given in supplement 2. There were several reasons for not re-assessing these participants that included transfer to another healthcare facility, leave of absence, unavailability on scheduled re-assessment days, and refusal to participate.

**Table 1:** Baseline characteristics of training participants

	7-day, n (%)	11-day, n (%)	Total, n (%)
Gender			
Male Female	36 (62.1) 22 (37.9)	29 (63.0) 17 (37.0)	65 (62.5) 39 (37.5)
Cadre			
Doctors Other providers	46 (79.3) 12 (20.7)	37 (80.4) 9 (19.6)	83 (79.8) 21 (20.2)
Other providers			
RN	4 (6.9)	0 (0.0)	4 (3.8)
LHV	3 (5.2)	1 (2.2)	4 (3.8)
Dispenser	4 (6.9)	4 (8.7)	8 (7.7)
HT	1 (1.7)	3 (6.5)	4 (3.8)
Midwife	0 (0.0)	1 (2.2)	1 (0.9)
Sector			
Public sector	50 (86.2)	41 (89.1)	91 (87.5)
Private sector	8 (13.8)	5 (10.8)	13 (12.5)
District			
Rawalpindi/Islamabad	23 (39.7)	24 (52.2)	47 (45.2)
Khairpur/Sukkur	35 (60.3)	22 (47.8)	57 (54.8)
Total	58 (100)	46 (100)	104 (100)

*RN* registered nurse, *LHV* lady health visitor, *HT* health technician

**Table 2:** Characteristics of participants re-assessed 6 months after training

	7-day, n (%)	11-day, n (%)	Total, n (%)
Gender			
Male	19 (65.5)	22 (68.8)	41 (67.2)
Female	10 (34.5)	10 (31.3)	20 (32.8)
Cadre			
Doctors	22 (75.9)	24 (75.0)	46 (75.4)
Other providers	7 (24.1)	8 (25.0)	15 (24.6)
Other providers			
RN	3 (10.3)	0 (0.0)	3 (4.9)
LHV	1 (3.4)	1 (3.1)	2 (3.3)
Dispenser	3 (10.3)	4 (12.5)	7 (11.5)
HT	0 (0.0)	3 (9.4)	3 (4.9)
Midwife	0 (0.0)	0 (0.0)	0 (0.0)
District			
Rawalpindi/Islamabad	13 (44.4)	16 (50.0)	29 (47.5)
Khairpur/Sukkur	16 (55.2)	16 (50.0)	32 (52.5)
Total	29 (100)	32 (100)	61 (100)

The 11-day training group had higher scores for knowledge and clinical skills in the pre-training and post-training assessments. In the re-assessment 6 months after training, the 11-day group performed better in the knowledge domain only (Table 3). However, the two groups had no statistically significant differences in the improvement and retention of knowledge and clinical skills (Table 4).

**Table 3:** Comparison of knowledge and clinical skills scores between the standard and abridged training groups

	7-day, mean (SD)	11-day, mean (SD)	*P* value
Knowledge			
Pre-training	49.2 (20.1)	60.7 (15.0)	0.019
Post-training	80.7 (11.8)	90.1 (6.9)	0.001
Re-assessment	68.3 (19.7)	83.7 (9.0)	0.001
Clinical skills			
Pre-training	44.8 (9.9)	51.3 (10.4)	0.018
Post-training	68.5 (7.7)	74.3 (6.6)	0.004
Re-assessment	62.2 (11.3)	65.2 (5.8)	0.236

**Table 4:** Improvement and retention of knowledge and clinical skills in the standard 11 days and 7 days abridged training groups

	7-day			11-day			*P* value
	n	Mean (SD)	95% CI	n	Mean (SD)	95% CI	
Improvement							
Knowledge	27	31.6 (18.4)	24.3–38.8	30	29.4 (14.8)	23.9–34.9	0.630
Clinical skills	26	23.8 (11.0)	19.3–28.2	31	23 (11.0)	18.9–27.0	0.784
Retention							
Knowledge	27	− 12.4 (15.2)	− 18.5–6.4	30	− 6.4 (10.9)	− 10.5–2.3	0.094
Clinical skills	26	− 6.3 (12.4)	− 11.3–1.3	31	− 9.1 (6.7)	− 11.5–6.6	0.308

The 11-day group scored higher in the post-training and re-assessment knowledge tests specifically for diarrhea, malaria, ARI, and meningitis. For sore throat and mastoiditis, the 11-day training group performed better in the post-training and re-assessment tests respectively (Table 5).

**Table 5:** Comparison of knowledge scores and clinical skills by health conditions between the standard 11 days and abridged 7 days training groups

Assessment domain	Post-training scores, mean (SD)		Re-assessment scores, mean (SD)	
Knowledge	7-day, n = 27	11-day, n = 30	7-day, n = 27	11-day, n = 30
General health	81.5 (21.4)	74.4 (16.8)	72.8 (24.5)	82.8 (22.5)
Possible bacterial infection (PBI)	92.6 (26.7)	100 (0)	92.6 (26.7)	100 (0)
Diarrhea	86.7 (15.7)^¥^	98 (8.1)^¥^	77 (24)^¥^	94 (10.7)^¥^
Malaria	74.1 (25.5)^*^	96.7 (12.7)^*^	59.3 (36.8)^¥^	86.7 (22.5)^¥^
Eye infection	88.9 (32)	96.7 (18.3)	48.1 (50.9)	63.3 (49)
ARI	77.8 (16.7)^¥^	87.5 (12.5)^¥^	64.8 (24.2)^¥^	77.2 (15.5)^¥^
Meningitis	77.8 (42.4)^¥^	96.7 (18.3)^¥^	63 (49.2)^¥^	96.7 (18.3)^¥^
Mastoiditis	81.5 (39.6)	96.7 (18.3)	63 (49.2)^¥^	96.7 (18.3)^¥^
Sore throat	81.5 (39.6)^¥^	100 (0)^¥^	77.8 (42.4)	83.3 (37.9)
Counseling	70.4 (46.5)	86.7 (34.6)	81.5 (39.6)	90 (30.5)
Otitis media	75.9 (29)	71.7 (25.2)	44.4 (16)	55 (24)
Clinical skills	7-day, n = 26	11-day, n = 31	7-day, n = 26	11-day, n = 31
Possible bacterial infection (PBI)	68.6 (15.1)	70.4 (15.3)	59.6 (15.8)	56.5 (13.4)
ARI	60.6 (16.9)	71.2 (11.8)	51.9 (17.4)	50.5 (12.7)
Diarrhea	85.9 (10.7)	82.1 (8.9)	82.9 (11)^¥^	91 (10.5)^¥^
Malaria	42.3 (27.2)^¥^	61.3 (33.4)^¥^	34.6 (30.9)	33.9 (23.8)
Otitis media	96.2 (19.6)	96.8 (18)	80.8 (40.2)^¥^	100 (0)^¥^
Malnutrition	56.4 (24.5)^¥^	72 (17.4)^¥^	59 (19.6)^¥^	73.1 (15.9)^¥^

*ARI* acute respiratory infection

^¥^*p* < 0.05

^*^p < 0.001

In the clinical skills assessment, the groups had similar scores for PBI and ARI in both tests. However, the 11-day group had significantly higher scores for questions on malnutrition in both post-training and re-assessment tests. For malaria, the 11-day group performed better in the immediate post-training assessment, while for diarrhea and otitis media, the 11-day group scored higher in the re-assessment only (Table 5).

The average cost of training was approximately US $75.00 per participant per day. This means that a total of US $825.00 was required for each participant to complete the standard 11-day IMNCI training compared to US $525.00 per participant for the 7-day training.

## Discussion

This study aimed to evaluate the effectiveness of an abridged IMNCI case-management training in a resource-limited country and to compare it with the standard 11-day training. The results of the study showed that while the two groups differed at baseline in their level of knowledge and their clinical skills proficiency, there were no statistically significant differences in the improvement and retention of knowledge and skills between the two groups. In contrast, a systematic review concluded that the standard IMCI training was more effective than its shorter versions, though the extent of this difference may be small (Rowe et al., 2012). Additionally, the lack of first-tier study designs directly comparing short and standard courses in literature was found to be a major limitation to definitively declaring the standard training as the superior option. However, since then, other studies have shown shorter courses to be as effective as the standard course. A study from Afghanistan showed that healthcare workers receiving a 7-day training performed equally well as workers that received the 11-day training (Mayhew et al., 2015). Another study demonstrated that nurses trained for 6 days in Rwanda had lower odds of incorrectly classifying fever and treating pneumonia than nurses trained for 11 days (Harerimana et al., 2014). India, from the onset, implemented a revised 8-days version of the WHO recommended 11-day course (Ingle & Malhotra, 2007). In 2012, a cluster-randomized trial demonstrated a decline in the infant mortality rate and the neonatal mortality rate for children born at home after the execution of the country’s 8-day version of the IMCI strategy (Bhandari et al., 2012). Moreover, two studies in India compared the outcomes of a further shortened interrupted 5-day training with the country’s standard 8-day training in primary healthcare workers. They demonstrated that the increase in assessment scores after training and the subsequent decline 3 years later were not statistically different between the two groups (Kumar et al., 2009; Venkatachalam et al., 2011).

We also found that the two groups did not perform consistently when the scores were evaluated for each individual illness. The overall knowledge scores for general health, PBI, eye infection, otitis media, and counseling were similar in both groups. Similarly, the clinical skills scores for PBI and ARI did not differ statistically both in the post-training and re-assessment tests. This indicates that the two training sessions were equally effective. However, the 11-day group scored higher on questions about sore throat, diarrhea, malaria, ARI, and meningitis in the post-training and re-assessment evaluations. We also observed that the 11-day group had higher scores in the clinical skills assessments for malnutrition, diarrhea, and otitis media at re-assessment. This finding may suggest that improving competency in the IMNCI approach for certain diseases may require additional time and attention during the training. Therefore, any measures for shortening the training should take this factor into consideration.

The standard training was considerably more costly, representing a 57% increase in expenses over the abridged training. The surplus funds can be reallocated to train more providers or for supervisory visits for previously trained providers. This reallocation can further improve the quality of care and sustain the quality of skills (Pariyo et al., 2005), as lack of long term supervision has been identified as an important obstacle to the proper implementation of IMCI (Reñosa et al., 2020).

The findings of the current study add to the existing literature where few studies have directly compared two training sessions of different lengths. To our knowledge, this is the first study in Pakistan that has developed, administered, and evaluated the effectiveness of an abridged IMNCI training module. The study also re-assesses the participants at 6 months to provide a more comprehensive picture of the effectiveness of the two training courses.

The study however had some limitations. First, it was carried out in 3 districts of Pakistan, so the findings may not be generalized. Second, the two groups participating in the study were not randomized. Nonetheless, a conscious effort was made to match the participants as much as possible.

## Conclusion

The improvement and retention of knowledge and clinical skills of healthcare providers receiving an abridged 7-day training are equivalent to providers receiving the standard 11-day training. However, knowledge and clinical skills for some illness may require increased allocated time. Additionally, the abridged training incurs fewer upfront costs than the standard training. Our study provides information about the effectiveness of scaling up an abridged IMNCI training program across the country to improve child health.

## Key Points

- The 7-day IMCI training is just as effective in improving and retaining clinical knowledge and skills as the 11-day training
- The 7-day training is more feasible due to lower cost
- However, proficiency in certain disease management was higher in the 11-day course, so more time may be required for training healthcare providers on management of these illnesses.

## Supplementary Information

Below is the link to the electronic supplementary material.

Supplementary file1 (DOCX 16 kb)

Supplementary file1 (DOCX 15 kb)
