# Supplementary material for: Evaluation the Effectiveness of Abridged IMNCI (7-Day) Course v Standard (11-Day) Course in Pakistan
Source: Matern Child Health J. 2021 Oct 20;26(3):530–6. doi: 10.1007/s10995-021-03276-3 (PMC8917018; doi:10.1007/s10995-021-03276-3)
Supplement: Supplementary file 1 — Supplementary file1 (DOCX 16 kb) [file 10995_2021_3276_MOESM1_ESM.docx]

**Supplementary file 1. Summary of abridged and standard IMNCI case management training**

| **Activity** | **7-day** | **11-day** |
| --- | --- | --- |
| **Registration** | 15 min | 30 min |
| **Opening presentation** |  | 1 hr |
| **Module 1. Introduction** | 30 min | 1 hr |
| **Module 2. Assess and Classify the Sick Child Age 2 Months up to 5 years** |  |  |
| Assess Diarrhea | 5 hrs | 5.5 hrs |
| Assess Fever | 2 hrs 45 min | 4 hrs |
| Classify Ear Problem | 2 hrs | 4 hrs |
| Video: General danger signs, cough or difficulty breathing |  |  |
| Outpatient session: Assess and classify the sick child:  Check for general danger signs  Assess and classify cough or difficulty breathing | 2 hrs | 4 hrs |
| Outpatient session:  Assess and classify diarrhea | 2 hrs | 4 hrs |
| Outpatient session:  Assess and classify fever | 2 hrs | 4 hrs |
| Outpatient and inpatient session:  Assess and classify ear problem  Check for malnutrition and anemia | 2 hrs | 4 hrs |
| **Module 3. Identify Treatment** | 3 hrs | 4 hrs |
| Outpatient session:  Assess and classify malnutrition and anemia | 2 hrs | 2 hrs |
| **Module 4. Treat the Child** | 4 hrs | 6 hrs |
| Teach Mother to Treat Local Infections | 2 hrs 30 min | 4 hrs |
| Outpatient session: Identify Treatment – Treat the Child:  Teach the mother to give oral drugs  Advise mother when to return immediately  Assess and classify sick children | 2 hrs | 4 hrs |
| Outpatient session: Treat the Child:  Plan A: Treat diarrhea at home  Plan B: Treat some dehydration with ORS  Plan C: Treat severe dehydration quickly  Assess and classify additional children | 2 hrs | 4 hrs |
| **Module 5. Counsel the mother** |  |  |
| Counsel the Mother About Feeding Problems | 2 hrs | 4 hrs |
| Outpatient session: Counsel the mother:  Counsel the mother about feeding problems  Observe and practice Plan B and Plan C  Assess and classify additional children | 2 hrs | 4 hrs |
| **Module 6. Management of the Sick Young Infant** |  |  |
| Classify Diarrhea | 2 hrs | 2 hrs |
| Video: Assess and classify young infant for bacterial infection |  |  |
| Outpatient and inpatient session: Management of the Sick Young Infant:  Assess and classify young infants for bacterial infection and diarrhea | 2 hrs 30 min | 4 hrs |
| **Module 7. Finish Management of the Sick Young Infant** | 4 hrs | 4 hrs |
| Video: Assessment of breastfeeding – positioning and attachment |  |  |
| Outpatient session: Management of the Sick Young Infant:  Assess breastfeeding  Correct positioning and attachment  Inpatient session:  Assess breastfeeding  Assess and classify young infants | 2 hrs 30 min | 4 hrs |
| **Module 8. Follow-up** | 2 hrs | 3 hrs |
| **Closing** | 1 hr | 1 hr |
