# Supplementary material for: Evaluation the Effectiveness of Abridged IMNCI (7-Day) Course v Standard (11-Day) Course in Pakistan
Source: Matern Child Health J. 2021 Oct 20;26(3):530–6. doi: 10.1007/s10995-021-03276-3 (PMC8917018; doi:10.1007/s10995-021-03276-3)
Supplement: Supplementary file 2 — Supplementary file1 (DOCX 15 kb) [file 10995_2021_3276_MOESM2_ESM.docx]

**Supplementary file 2. Characteristics of participants who were not re-assessed at 6 months**

| **Characteristics** | **7-day, n (%)** | **11-day, n (%)** | **Total, n (%)** |
| --- | --- | --- | --- |
| **Gender** |  |  |  |
| Male  Female | 17 (58.6)  12 (41.4) | 7 (50.0)  7 (50.0) | 24 (55.8)  19 (44.2) |
| **Cadre** |  |  |  |
| Doctors  Other providers | 24 (82.8)  5 (17.2) | 13 (92.9)  1 (7.1) | 37 (86.0)  6 (14.0) |
| **Other providers** |  |  |  |
| RN  LHV  Dispenser  HT  Midwife | 1 (3.4)  2 (6.9)  1 (3.4)  1 (3.4)  0 (0.0) | 0 (0.0)  0 (0.0)  0 (0.0)  0 (0.0)  1 (7.1) | 1 (2.3)  2 (4.6)  1 (2.3)  1 (2.3)  1 (2.3) |
| **District** |  |  |  |
| Rawalpindi/Islamabad  Khairpur/Sukkur | 10 (34.5)  19 (65.5) | 8 (57.1)  6 (42.9) | 18 (41.9)  25 (58.1) |
| **Total** | **29 (100)** | **14 (100)** | **43 (100)** |

RN; registered nurse, LHV; lady health visitor, HT; health technicians
